# A Textbook Case of Human T-lymphotropic Virus-1 (HTLV-1)-Induced Adult T-cell Leukemia Treated With Cyclophosphamide, Hydroxydaunorubicin, Oncovin, and Prednisone/Prednisolone (CHOP)

**DOI:** 10.7759/cureus.49169

**Published:** 2023-11-21

**Authors:** Faryal Altaf, Zaheer A Qureshi, Sarah Moore, Tiffany-Marie Golek, Arpan Chawala

**Affiliations:** 1 Department of Internal Medicine, BronxCare Health System, Bronx, USA; 2 Department of Internal Medicine, The Frank H. Netter M.D. School of Medicine at Quinnipiac University, Bridgeport, USA; 3 Department of Internal Medicine, St. Vincent's Medical Center, Bridgeport, USA; 4 Department of Medicine, American University of the Caribbean School of Medicine, Cupecoy, SXM

**Keywords:** hypercalcemia in atll, htlv-1-associated atll, adult t-cell leukemia-lymphoma (atll), general internal medicine, oncology, hematology, infective dermatitis, strongyloides stercoralis, human t-lymphotropic virus (htlv), adult t-cell lymphoma/leukemia (atl)

## Abstract

Human T-lymphotropic virus-1 (HTLV-I) is an enveloped, single-stranded RNA virus of the Retroviridae family. The virus causes two well-recognized disease associations: adult T-cell leukemia/lymphoma (ATL) and HTLV-I-associated myelopathy (HAM), also known as tropical spastic paraparesis (TSP). We report a case of HTLV-1-induced adult T-cell lymphoma/leukemia in a 45-year-old female who presented with complaints of swelling on the right side of her neck and rash on her upper and lower extremities and abdomen. The patient also had a history of strongyloidiasis infection and Crohn's disease. She was found to have hypercalcemia and multiple lytic lesions of the bone found on the imaging. She also tested positive for HTLV-1 and T cell-positive for cluster of differentiation (CD) 2, CD3, partial CD5, and minimal CD56, later confirmed by the bone marrow (BM) and skin punch biopsies. ATL is characterized by the clonal proliferation of CD4+ T cells containing randomly integrated HTLV-I provirus, often associated with T-cell receptor gene rearrangements. ATL, in its aggressive forms, has one of the poorest prognoses of non-Hodgkin lymphoma. It is essential to raise awareness of ATL, although further research and trials are needed to solidify the treatment options to prevent mortality.

## Introduction

Adult T-cell lymphoma (ATL) has four types depending on the presentation: acute, lymphomatous, chronic, and smoldering [1]. Acute's clinical features include skin lesions (nodules, ulcers, and generalized widespread rash), lytic bone lesions, hypercalcemia, pulmonary infiltrates with elevated serum cluster of differentiation (CD) 25 (interleukin 2 receptor), and serum thymidine kinase activity, and serum neuron-specific enolase lymphomatous' clinical features include lymphadenopathy, hepatosplenomegaly, and skin lesions [2]. Hypercalcemia does not occur in the chronic type. Smoldering presented with skin and pulmonary lesions does happen, but other clinical features such as lymphadenopathy, hepatosplenomegaly, and hypercalcemia are absent [3]. Globally, human T-lymphotropic virus-1 (HTLV-1) infects 20 million people with ATL 0.05 per 100,000 [1,2]. In the United States, the incidence of ATL is approximately 0.05 per 100,000 [1-3].

## Case presentation

A 45-year-old female from Ghana (recently visited in 2021 and immigrated in 2017) with a past medical history of hypertension, prediabetes, lumbar spine radiculopathy with disc herniation, Crohn's disease, tension headaches, strongyloidiasis, *Helicobacter pylori* infection, and asymptomatic thymic enlargement presented with a papular, painful, and pruritic rash for one month. The rash started on the back of her hands and extended to the feet, extensor surfaces, abdomen, and buttocks. The patient visited her primary care physician two weeks ago, and oral allergy medications plus topical medications were prescribed along with the discontinuation of gabapentin, but that did not help the rash. The patient endorsed nausea and vomiting for the past week, weight loss of an unknown amount, and right-sided neck swelling with tenderness for six months. She denied smoking, drinking, or illicit drug use. The patient was febrile and hemodynamically stable, and the laboratory results of the initial presentation are mentioned in Table 1.

**Table 1:** Initial laboratory results INR, international normalized ratio; LDH, lactate dehydrogenase; HDL, high-density lipoprotein

Laboratory Test	Patient Values	Reference Range
Hemoglobin (Hb) (g/dL)	12	12.0-16.0
Hematocrit (%)	37.2	42.0-51.0
Eosinophil Count (k/μL)	0.00	0.05-0.25
Erythrocyte Sedimentation Rate (mm/hour)	53.0	≤20
Prothrombin Time (PT) (second{s})	17.6	9.9-13.3
INR	1.47	1.14
Sodium (mEq/L)	133	135-145
Serum Calcium (mg/dL)	14.0	8.5-10.5
Alanine Aminotransferase (U/L)	57	5-40
Aspartate Aminotransferase (U/L)	59	9-36
Creatine Kinase (U/L)	18	20-200
Alkaline Phosphatase (U/L)	234	42-98
LDH (U/L)	512	100-190
C-reactive Protein (mg/L)	97.71	≤5.00
Ceruloplasmin (ng/mL)	395.0	13.0-150.0
Haptoglobin (mg/dL)	307.0	30.0-200.0
HDL Cholesterol (mg/dL)	29	34-82
Vitamin B12 (pg/mL)	>1999	243-894
Vitamin D 25-OH (ng/mL)	28.8	30.0-100.0

**Figure 1:**
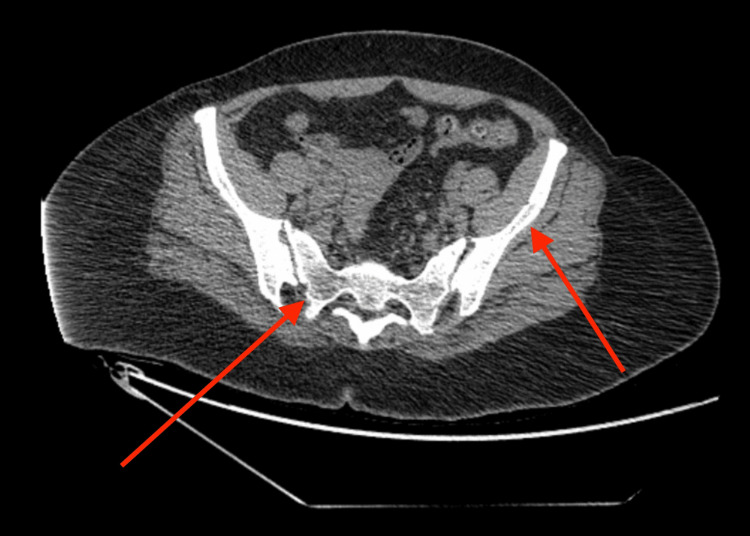
CT of the abdomen and pelvis showing bilateral iliac lymphadenopathy and erosive lesions of the spine and iliac bones The red arrows show erosive lesions CT: computed tomography

On physical examination, the neck was noted to have tenderness and mobile anterior cervical right-sided lymph nodes. The rash was raised with hyperpigmentation and scaling. A computed tomography (CT) of the abdomen and pelvis revealed bilateral iliac and groin lymphadenopathy and widespread lytic bone lesions, suggesting widespread bony metastatic disease, as shown in Figure 1. A CT of the chest without contrast revealed supraclavicular lymphadenopathy with the right worse than the left and multiple pulmonary nodules, the largest being 3 mm in the upper right lobe. Laboratory work revealed lactic acidosis of 2.3 mmol/L (normal: <2 mmol/L) and hypercalcemia with serum calcium (Ca) of 14 mg/dL (normal: 8.6-10.3 mg/dL). Based on laboratory and CT findings, the patient was admitted to the intensive care unit. Endocrinology was consulted for hypercalcemia, who recommended starting aggressive crystalloid fluids, and if there was no response, then later, calcitonin could be used. The patient's calcium improved on the intravenous (IV) fluids, and calcitonin was unnecessary. The patient started on the topical ointment diflorasone for rash by dermatology. No significant improvement was seen with the use of diflorasone. Laboratory tests were sent for HTLV, flow cytometry, and serum protein electrophoresis (SPEP). SPEP and urine protein electrophoresis were nonspecific.

**Figure 2:**
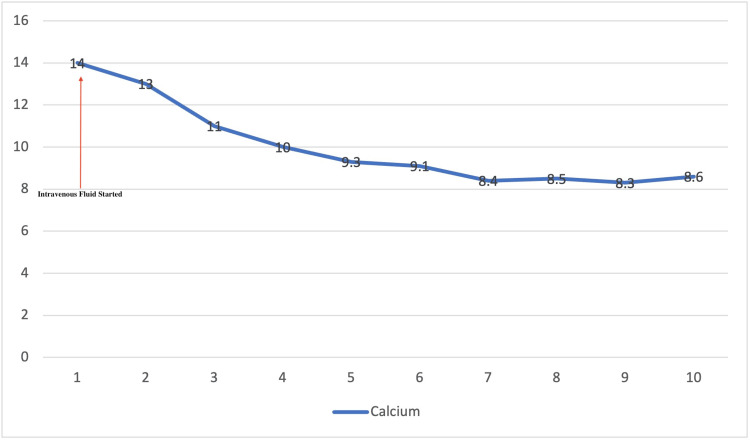
Trend of serum calcium The x-axis shows the days of the hospital course. The y-axis shows serum calcium in mg/dL

**Figure 3:**
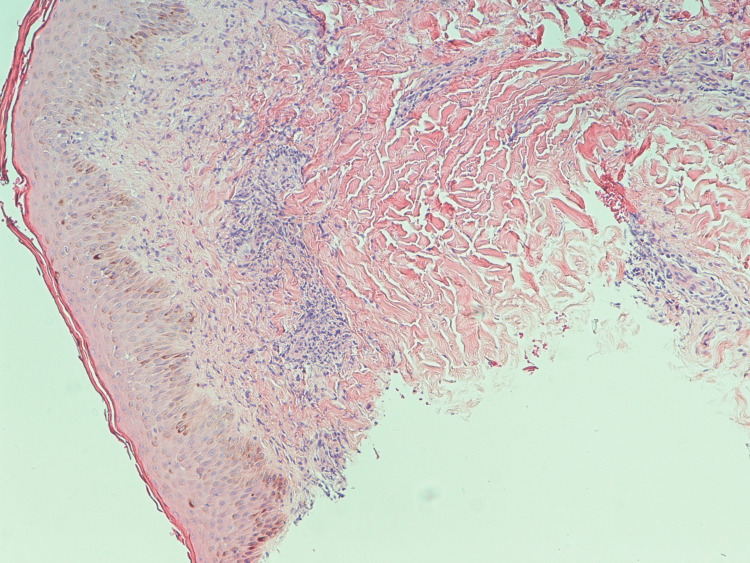
The H&E-stained section shows lymph node with immunomorphologic findings consistent with peripheral T-cell lymphoma The H&E-stained section shows lymph node with the complete effacement of the nodal architecture by a diffuse polymorphous infiltrate of small lymphocytes, eosinophils, histiocytes, and frequent medium-to-large-sized atypical lymphoid cells with irregular nuclei, variably prominent nucleoli, and clumped-chromatin. Scattered large atypical lymphoid cells with Hodgkin and Reed-Sternberg (HRS) cell-like morphology are noted. Increased mitotic activity is seen. Cluster of differentiation (CD) 7-, CD4(variable)+, CD8-, CD25+, CD10-, PD1-/+, BCL6-/+, TBET-, GATA3+/-, FOXP3-/+, MUM1+/-, CD30-, CD45(normal to weak)+, and EBER-. The ki67 proliferation index is high. Small B-cells (CD20+ and PAX5+) are mostly in the follicles. Small CD8+ T cells are seen scattered. The large HRS-like cells show the following immunophenotype: PAX5(weak)+, CD20(variable)+, MUM1+, CD30+, CD45(variable)+, CD15-, CD3-, CD5-, and EBER-. CD10 stains stromal elements. CD21 highlights residual follicular dendritic meshwork, and CD34 shows no increase in vessels. The morphologic and immunomorphologic findings are consistent with peripheral T-cell lymphoma H&E, hematoxylin and eosin; PD-1, programmed cell death protein 1; BCL6, B-cell lymphoma 6; TBET, T-box expressed in T cells; GATA3, GATA binding protein 3; FOXP3, forkhead box P3; MUM1, multiple myeloma oncogene 1; EBER, Epstein-Barr virus expressing mRNA; PAX5, paired-box gene 5

The patient underwent a right-sided supraclavicular lymph node excision biopsy and a punch biopsy of the skin on the abdominal wall. The patient was started on intravenous (IV) crystalloid fluids for hypercalcemia, and calcium started to trend down, as shown in Figure 2.** **The flow cytometry shows atypical T-cell populations expressing CD2, CD3, partial CD5, and minimal CD56. Lymph node biopsy revealed the complete effacement of nodal architecture with a diffuse polymorphous infiltrate, atypical lymphoid cells with irregular nuclei, Hodgkin and Reed-Sternberg cell-like morphology, and increased mitotic activity, as shown in Figure 3. Bone marrow (BM) biopsy revealed large pleomorphic lymphocytes, rare erythroid and myeloid precursors, and atypical lymphocytes. The morphologic and immunomorphologic findings were consistent with peripheral T-cell lymphoma. The patient's clinical condition improved, and she was transferred to a tertiary care center for cyclophosphamide, hydroxydaunorubicin (Adriamycin), Oncovin (vincristine), and prednisone/prednisolone (CHOP) induction, interferon-based therapy, and bone marrow transplant. The patient stayed on CHOP for six months as per the oncology recommendation. And patient showed near-complete resolution of symptoms, including rash and weakness.

## Discussion

Human T-lymphotropic virus-1 (HTLV-1) is a CD4+T retrovirus. It was first discovered by the Gallo in 1979 [4]. A set of patients established by J. Minna and A. Gazdar became the first documented detection of HTLV-1 [4]. These patients exhibited the virus' rare yet lethal sequelae, cutaneous T-cell lymphoma [4]. There are currently seven known subtypes of HTLV-1, A-F, containing several strains originating in various African and Asian countries [5-7]. While this virus is considered a rising epidemic and infects the host indefinitely, the virus typically yields little immunosuppression to those infected [8,9]**. **Approximately 95% of individuals who contract the virus remain lifelong asymptomatic carriers [7-9]. The remaining 5% of those with HTLV-1 may succumb to malignancies, inflammatory or opportunistic pathology [8,9].

The major routes of viral transmission include vertical transmission, sexual transmission, and transmission via blood transfusion [10]. While sexual contact is the most common mode of transmission, vertical transmission in childhood is a significant factor in developing associated adult T-cell lymphoma [11,12]. Vertical transmission from breast milk increases the risk for the transmission of HTLV-1 to a child by four times, a substantial margin [13]. Several clinical manifestations coincide with HTLV-1 infection. One example is infective dermatitis (ID). ID is considered vertical transmission-dependent, as the onset begins before two years of age and resolves before adulthood. ID is not a manifestation of HTLV-1 isolated to childhood, as emerging cases of adult-onset ID associated with HTLV-1 infection have been recently described [14-17].

Strongyloidiasis infection is strongly associated in those with symptomatic associations with the HTLV-1 virus. *Strongyloides*' infective course is either asymptomatic or mild in the healthy population [18]. The danger lies in the development of autoinfection, where larvae enter the bloodstream and disseminate throughout the body [18]. The HTLV-1-infected individual lies vulnerable to autoinfection due to a decrease in native anti-helminthic protective measures of the immune system, leading to a substantial increase in the frequency of disseminated *Strongyloides* infection among HTLV-1-infected individuals [19].

Adult T-cell lymphoma caused by HTLV-1 and its subtypes are rare neoplasms with CD4+ CD25+ T-cell markers [20]. This lymphoma/leukemia is aggressive and can carry a grimmer prognosis than its non-Hodgkin lymphoma counterparts [21]. Due to the heterogeneity in the profiles of associated adult T-cell lymphoma, the malignancy is further divided into four types of subcategories: acute, lymphoma, chronic, and smoldering. Acute is regarded as the more aggressive of the four subtypes [22]. Furthermore, paraneoplastic hypercalcemia is one of the most common and most lethal consequences of adult T-cell lymphoma. The proposed mechanism of hypercalcemia is due to inappropriate bone resorption via excessive osteoclast accumulation [23]. Adult T-cell lymphoma-associated hypercalcemia is a consequence of several cytokine activations such as interleukin 1 and transforming growth factor-β [24,25]. Moreover, the inappropriate expression of parathyroid hormone-related protein (PTHrP), while not consistently increased in adult T-cell lymphoma-associated hypercalcemia, plays a fundamental role in adult T-cell lymphoma-associated hypercalcemia, particularly in immunodeficient mice models implanted with leukemic cells [26]. Serum calcium levels are increased in approximately 70% of HTLV-1-positive individuals with adult T-cell lymphoma, and it is one of the most significant increases in mortality within this patient population [26].

ATL diagnosis is based on clinical features, characteristics, and morphologic and immunophenotypic changes of malignant cells [27]. Ideally, the identification of a minimum of 5% of tumor cells in peripheral blood and the confirmation of HTLV-1 in the blood are sufficient for diagnosis. The lymph node biopsy can sometimes add to the diagnosis [28]. The current treatment modalities of ATL are based on subtypes and responses to initial therapy. It differs across countries as well due to the nonavailability of therapy modalities. Treatment includes multiagent chemotherapy, zidovudine plus interferon alpha, and allogeneic hematopoietic stem cell transplantation [27,29]. Treatment options also include the CHOP and rituximab (R)-CHOP regimens [30]. Pralatrexate is also under investigation [31]. In 2021, mogamulizumab was recently approved for refractory ATL in Japan. Brentuximab vedotin is not yet FDA-approved, but its use in CD30+ cases may benefit research [31].

## Conclusions

In the case of the patient presented, she offered several pathological correlates, including dermatologic manifestations, hypercalcemia, and previous strongyloidiasis infection. ATL, in its aggressive forms, carries one of the poorest prognoses in non-Hodgkin lymphoma. This case report illustrates the diverse complexity of patients with ATL. It is one of the few cancers that are caused by a virus. The findings are only sometimes typical. Recent advances in oncology and hematology have shown promising results for mogamulizumab and lenalidomide. As physicians, we should keep a low threshold for these diagnoses. It is essential to raise awareness of ATL, although further research and trials are needed to solidify the treatment options to prevent mortality.

## References

[1] Kaplan JE, Osame M, Kubota H (1990). The risk of development of HTLV-I-associated myelopathy/tropical spastic paraparesis among persons infected with HTLV-I. J Acquir Immune Defic Syndr (1988).

[2] Cleghorn FR, Manns A, Falk R (1995). Effect of human T-lymphotropic virus type I infection on non-Hodgkin's lymphoma incidence. J Natl Cancer Inst.

[3] Kondo T, Kono H, Miyamoto N (1989). Age- and sex-specific cumulative rate and risk of ATLL for HTLV-I carriers. Int J Cancer.

[4] Gallo RC (2005). The discovery of the first human retrovirus: HTLV-1 and HTLV-2. Retrovirology.

[5] Slattery JP, Franchini G, Gessain A (1999). Genomic evolution, patterns of global dissemination, and interspecies transmission of human and simian T-cell leukemia/lymphotropic viruses. Genome Res.

[6] Mahieux R, Ibrahim F, Mauclere P (1997). Molecular epidemiology of 58 new African human T-cell leukemia virus type 1 (HTLV-1) strains: identification of a new and distinct HTLV-1 molecular subtype in Central Africa and in Pygmies. J Virol.

[7] Salemi M, Van Dooren S, Audenaert E, Delaporte E, Goubau P, Desmyter J, Vandamme AM (1998). Two new human T-lymphotropic virus type I phylogenetic subtypes in seroindeterminates, a Mbuti pygmy and a Gabonese, have closest relatives among African STLV-I strains. Virology.

[8] Asquith B, Bangham CR (2008). How does HTLV-I persist despite a strong cell-mediated immune response?. Trends Immunol.

[9] Bryan ES, Tadi P (2023). Human T-cell lymphotropic virus. https://www.ncbi.nlm.nih.gov/books/NBK560825/.

[10] Tsukasaki K (2012). Adult T-cell leukemia-lymphoma. Hematology.

[11] Steglich RB, Tonoli RE, Souza PR, Pinto GM, Riesgo Rdos S (2015). HTLV-1-associated infective dermatitis and probable HTLV-1- associated myelopathy in an adolescent female. An Bras Dermatol.

[12] Paiva AM, Assone T, Haziot ME (2018). Risk factors associated with HTLV-1 vertical transmission in Brazil: longer breastfeeding, higher maternal proviral load and previous HTLV-1-infected offspring. Sci Rep.

[13] Bittencourt AL, de Oliveira Mde F (2010). Cutaneous manifestations associated with HTLV-1 infection. Int J Dermatol.

[14] Lee R, Schwartz RA (2011). Human T-lymphotrophic virus type 1-associated infective dermatitis: a comprehensive review. J Am Acad Dermatol.

[15] Manns A, Hisada M, La Grenade L (1999). Human T-lymphotropic virus type I infection. Lancet.

[16] de Oliveira Mde F, Vieira Md, Primo J (2010). Flower cells in patients with infective dermatitis associated with HTLV-1. J Clin Virol.

[17] Carvalho EM, Da Fonseca Porto A (2004). Epidemiological and clinical interaction between HTLV-1 and Strongyloides stercoralis. Parasite Immunol.

[18] Pays JF (2011). [Combined infection with HTLV-1 and Strongyloides stercoralis] (Article in French). Bull Soc Pathol Exot.

[19] Mehta-Shah N, Ratner L, Horwitz SM (2017). Adult T-cell leukemia/lymphoma. J Oncol Pract.

[20] Kiyokawa T, Yamaguchi K, Takeya M (1987). Hypercalcemia and osteoclast proliferation in adult T-cell leukemia. Cancer.

[21] Hagler KT, Lynch JW Jr (2004). Paraneoplastic manifestations of lymphoma. Clin Lymphoma.

[22] Fukumoto S, Matsumoto T, Ikeda K (1988). Clinical evaluation of calcium metabolism in adult T-cell leukemia/lymphoma. Arch Intern Med.

[23] Gibbs WN, Lofters WS, Campbell M (1987). Non-Hodgkin lymphoma in Jamaica and its relation to adult T-cell leukemia-lymphoma. Ann Intern Med.

[24] Wano Y, Hattori T, Matsuoka M, Takatsuki K, Chua AO, Gubler U, Greene WC (1987). Interleukin 1 gene expression in adult T cell leukemia. J Clin Invest.

[25] Niitsu Y, Urushizaki Y, Koshida Y, Terui K, Mahara K, Kohgo Y, Urushizaki I (1988). Expression of TGF-beta gene in adult T cell leukemia. Blood.

[26] Takaori-Kondo A, Imada K, Yamamoto I, Kunitomi A, Numata Y, Sawada H, Uchiyama T (1998). Parathyroid hormone-related protein-induced hypercalcemia in SCID mice engrafted with adult T-cell leukemia cells. Blood.

[27] Phillips AA, Harewood JC (2018). Adult T cell leukemia-lymphoma (ATL): state of the art. Curr Hematol Malig Rep.

[28] Yamada Y, Tomonaga M, Fukuda H (2001). A new G-CSF-supported combination chemotherapy, LSG15, for adult T-cell leukaemia-lymphoma: Japan Clinical Oncology Group Study 9303. Br J Haematol.

[29] Taylor GP, Matsuoka M (2005). Natural history of adult T-cell leukemia/lymphoma and approaches to therapy. Oncogene.

[30] Marneros AG, Grossman ME, Silvers DN (2009). Pralatrexate-induced tumor cell apoptosis in the epidermis of a patient with HTLV-1 adult T-cell lymphoma/leukemia causing skin erosions. Blood.

[31] Subramaniam JM, Whiteside G, McKeage K, Croxtall JC (2012). Mogamulizumab: first global approval. Drugs.

